# Study protocol for measuring the impact of (quasi-)monochromatic light on post-awakening cortisol secretion under controlled laboratory conditions

**DOI:** 10.1371/journal.pone.0267659

**Published:** 2022-05-18

**Authors:** Sebastian Babilon, Paul Myland, Julian Klabes, Joel Simon, Tran Quoc Khanh

**Affiliations:** 1 Laboratory of Lighting Technology, Technical University of Darmstadt, Darmstadt, Germany; 2 Light and Health Research Center, Department of Population Health Science and Policy, Icahn School of Medicine at Mount Sinai, New York, NY, United States of America; University of Oxford, UNITED KINGDOM

## Abstract

Cortisol secretion has a fundamental role in human circadian regulation. The cortisol awakening response (CAR) can be observed as a daily recurring sharp increase in cortisol concentration within the first hour after awakening and is influenced by environmental light conditions. The current work provides the study protocol for an ongoing research project that is intended to explore the spectral dependencies and to discuss measures of emotional state and cognitive functioning potentially related to the CAR. Based on a controlled within-subjects sleep laboratory study, the impact of a two-hour, (quasi-)monochromatic, post-awakening light exposure of different peak wavelength (applied from 6:00 to 8:00 am) on resulting CAR levels should be investigated in a systematic manner to eventually derive a corresponding spectral sensitivity model. As a secondary outcome, it should be explored whether a potentially light-enhanced cortisol secretion might also impact different measures of sleepiness, mood, and vigilance for certain wavelengths. The study protocol described in the present work discusses the various protocol steps using pilot data collected for two different wavelength settings (i.e., short-wavelength blue-light at λ_max_ = 476 nm and long-wavelength red-light at λ_max_ = 649 nm) experienced by a group of four healthy male adults at an average ± SD age of 25.25 ± 3.59 years.

## Introduction

Cortisol, like melatonin, plays an important role in the human circadian system [1]. It is an essential hormone, synthesized by the cortex of the adrenal gland and regulated via the hypothalamic-pituitary-adrenal (HPA) axis [2–4], that is associated with waking, alertness, and stress response [5]. Such being the case, cortisol affects both cognitive and emotional networks of the central nervous system [6–10]. Its genuine secretion pattern shows a circadian rhythm with a maximum in the early morning hours [11, 12]. Throughout the day, its concentration in the body decreases, reaching a broad minimum in the evening, before rising again slowly during nighttime. As with other circadian rhythms, cortisol secretion is ultimately controlled by the hypothalamic suprachiasmatic nucleus (SCN) [13] and appears to be influenced by sleep [14–18] and the environmental light conditions [19, 20].

In addition to its fundamental circadian regulation, a sharp increase in cortisol concentration, known as the cortisol awakening response (CAR) [21–24], is observed within the first hour after awakening. Wilhelm *et al*. [25] provided evidence that this rise occurs in direct response to the transition from sleep to wake, superimposing the circadian oscillation of HPA axis activity. It is hypothesized that its magnitude is linked to the anticipation of stress [26, 27] so that a reduced CAR may result in a decreased capability to deal with external stressors [28]. In addition, CAR is assumed to play an important role in preparing (boosting) the individual for the upcoming day [29], e.g., by acting as a time-of-day marker to optimize cognitive function appropriately [30, 31].

In this context, morning light exposure has been shown to be capable of significantly enhancing CAR in both sleep-restricted and non-sleep-restricted humans. Scheer and Buijs [32] for example investigated the effect of polychromatic (white) light on the morning cortisol responses of fourteen male participants after a habitual night’s sleep at their homes. By exposing the subjects to 800 lx (at vertical eye level) for one hour using a light visor (BioBrite Inc., Bethesda, MD, USA), they were able to measure an increase in cortisol concentration of 35% compared to waking up in dim light only. Similar results were found by Leproult *et al*. [33] who investigated the alerting and endocrine effects of bright-light morning and afternoon exposure on a group of eight healthy, sleep-deprived men. Over the course of a 3-hours period, they applied in each case a dynamic change of illuminance in the range from 2000 to 4500 lx at the subjects’ vertical eye level using fluorescent tube luminaires. The resulting endocrine responses were eventually compared to those obtained for a static dim light control condition of less than 150 lx. While the afternoon exposure (13:00–16:00 h) compared to the dim light control did not show an impact on cortisol levels, a 50% increase was reported for the morning exposure (05:00–08:00 h) applying the same intervention scheme. Looking into the effects of dawn simulation (250 lx; Natural Alarm Clock, Outside In, Cambridge Ltd, UK) on the morning cortisol responses and mood states of a group of twelve healthy subjects (five females and seven males), Thorn *et al*. [34] quoted an average increase in the total cortisol release within the first 45 min after awakening of 12.8% compared to days on which no dawn simulation was used for wake-up.

Conversely, Choi *et al*. [35], who compared the awakening effects of 2-hours of blue-enriched (color temperature of 6500 K) and warm-white (color temperature of 3500 K) LED light exposure (09:00–11:00 h; 500 lx horizontal illuminance following ISO standard) on fifteen university students (seven females and eight males), found that both light conditions significantly reduced post-exposure salivary cortisol levels. No significant difference was found between the light conditions. In another study investigating the implications of a 6-hours bright light exposure (4100 K fluorescent light transmitted through a UV-stable filter; 10000 lx at the cornea) during the ascending and descending phases of the circadian cortisol secretion of twenty healthy subjects (five females and fifteen males), Jung *et al*. [36] found a significant decrease in cortisol levels during both phases, i.e., during the individual’s biological night and morning, respectively, compared to dim light exposure over the same time period. Finally, Touitou *et al*. [37] investigated the impact of a 2-hours bright light post-awakening exposure (2000 lx at eye level; Lumière du Jour, GAMAIN, Paris, France) on the endocrine responses of a group of six healthy, sleep-deprived male subjects. Sleep deprivation was achieved by initiating an early wake-up on experimental days that reduced the subjects’ habitual sleep schedule (23:00–07:00 h) by 2 h. Compared to a dim light control condition, no significant differences were found with regard to the means and magnitudes of the conducted morning cortisol measurements.

Thus, even though some inconsistencies between the research results can be identified, the existence of an overall stimulatory effect of (white/bright) light exposure is well-accepted among researchers [38]. However, little is still known about the spectral dependencies and how a light-induced increase (or decrease) of the CAR translates to performance and subjective post-awakening measures. In this context, the first study investigating the effects of a monochromatic light intervention on the morning cortisol secretion was performed by Figueiro and Rea [28] in a sleep laboratory environment. Upon awakening from a 4.5 h-restricted sleep opportunity, they exposed eighteen adolescents (equal gender ratio) to a blue/short-wavelength light stimulus (40 lx vertical at the subjects’ cornea; 470 nm peak wavelength) for a total duration of 80 min post-awakening (06:00–07:20 h) using LED goggles. In comparison to a dim light control session (incandescent light source, < 5 lx at the cornea), a significantly increased CAR was observed for the blue light intervention, indicating that morning exposure to moderate levels of short-wavelength light can have a strong stimulatory effect on humans. In a recently published article, Petrowski *et al*. [38] confirmed the spectral dependencies of light-induced effects on the morning cortisol response. In a sleep laboratory setting, they compared the impact of three different monochromatic stimuli (blue, 470–480 nm peak wavelength, 201 lx; green, 520 nm peak wavelength, 806 lx; red, 630 nm peak wavelength, 235 lx) on the post-awakening cortisol levels of a group of 23 healthy male adults. Following a 6-hours sleep opportunity (23:00–05:00 h), 1-hour of LED light exposure was applied starting 5 min after wake-up by using a half-moon integrating sphere. Cortisol levels were measured every 15 minutes up to 30 min after the light exposure has ended. From the analysis of these data, a statistically significant main effect of wavelength was reported. In addition, post-hoc tests revealed significantly higher overall cortisol levels for the blue and green light conditions than for the red light exposure, providing further evidence that light-induced effects on the morning cortisol response depend on the spectral composition of the light intervention.

From the studies discussed above, it is not yet possible to determine whether the known melatonin pathway [39–41] of retinal projections from a subset of melanopsin-expressing retinal ganglion cells (mRGCs) to the SCN is also involved in the CAR response to light exposure or if the spectral sensitivity of the HPA axis is identical with (or at least can be approximated by) the well-established action spectrum for melatonin regulation in humans [42, 43]. In addition, previous studies, if included in their analysis, failed to report a clear linkage between light-induced CAR enhancement and the potential improvement of post-awakening performance and subjective measures, such as for example mood state and reported sleepiness. Of the studies explicitly dealing with light-induced effects on CAR, only those of Leproult *et al*. [33] and Choi *et al*. [35] reported significant effects of bright or blue-enriched white light interventions on additional measures other than cortisol levels—still leaving us with an incomplete and only partly conclusive picture.

Thus, building up on previous studies, the present lab protocol aims at establishing a fundamental methodology that allows for a further exploration of the spectral dependencies of morning cortisol secretion in a consistent and expedient manner. For this purpose, a suitably designed within-subjects sleep laboratory study, which is intended to investigate the post-awakening effects of 2-hours of monochromatic light exposure for different wavelengths, will be presented. Pilot data of mean cortisol levels, mean reaction times as well as subjective mean ratings of sleepiness and mood state will eventually be shown for a blue-vs-red light exposure to illustrate the application of the protocol. Based on the findings of Petrowski *et al*. [38], it can be expected that the cortisol secretion is stronger for blue than for red light, which would indicate a higher sensitivity of the HPA axis in the short-wavelength regime. Moreover, it can be assumed that such an additional “boosting” provoked by an enhanced CAR may also be reflected in the remaining outcome measures. In particular, the ongoing study is among the first to explicitly consider the subjects’ reaction times in response to auditory stimuli to be potentially related to light-induced changes in CAR.

## Materials and methods

### Participants

To preclude sex differences as a potential confounding factor in the cortisol analysis, the pilot study was limited to male participants only as literature suggests that men and women show different cortisol responses to external stressors [44]. Thus, a search for suitable subjects was initiated among the university students through email notices, electronic and conventional postings, and word-of-mouth. Potential candidates were then invited to the laboratory for initial screening. They had to complete questionnaires about their sleep quality (Pittsburgh Sleep Quality Index, PSQI [45]), chronotype (Munich Chrono Type Questionnaire, MCTQ [46]), general health status (36-item Short-Form Health Survey, SF-36 [47]), and stress perception (10-item Perceived Stress Scale, PSS-10 [48]). Candidates were excluded if they showed a PSQI score of five or greater, were identified as extreme morning or evening types from the MCTQ, had scores on any of the SF-36 sub-scales that were at least one standard deviation (SD) smaller than the respective age- and gender-dependent mean values of the normative sample obtained from the German Federal Health Survey 1998 [49], or experienced recent episodes of intense stress indicated by a PSS-10 score of more than one SD larger than the age- and gender-dependent mean score of a representative sample of the German population reported by Klein *et al*. [50]. Further exclusion criteria comprise smoking, medication or drug consumption, excessive alcohol use or caffeine intake, history of chronic health problems, psychiatric, mental or sleep disorders, and shift work or transmeridian flights within the last two month prior to the screening day. Potential candidates were asked to indicate whether they had or experienced any of the above as part of a personal interview, in which they were also informed about the experimental procedure, their rights and obligations as a study participant, and the possible side effects of the experiment. In addition, candidates were screened for color deficiency by applying the Ishihara’s Tests for Colour Deficiency [51], the Standard Pseudoisochromatic Plates Part II for Acquired Color Vision Defects by Ichikawa *et al*. [52], and the Farnsworth-Munsell D-15 Color Vision Test [53]. They were excluded from the study in case that indication for any sort of color vision impairment was given. Candidates wearing glasses or contact lenses for visual acuity correction were not excluded from the study as long as the limits of ± 6 dpt or ± 4 dpt in case of astigmatism were not exceeded. However, they were asked to remove their optical aids before the beginning of each test session.

Based on the initial screening process, a total of four healthy men, who met all study criteria, were selected to participate in the experiments. Their average ± SD age was 25.25 ± 3.59 years. According to the guidelines of the German Federal Center for Health Education [54], all participants showed a low-risk alcohol drinking behavior (less than 24 g of pure ethanol per day for men). In addition, they were identified as low to moderate caffeine consumers according to Addicott *et al*. [55] (less than 405 mg of caffeine per day). All participants indicated to maintain a regular sleep schedule. Their average ± SD bed time was 11:08 pm ± 1.2 min with an average ± SD sleep onset of 15.63 ± 9.43 min, a regular average ± SD wake-up time of 6:53 am ± 1.4 min, and 7.03 ± 0.49 h of average ± SD effective sleep. The subjects were paid for taking part in the study. Informed consent was obtained from all participants. The study was approved by the Ethical Review Committee of the Technical University of Darmstadt (TU Darmstadt) on December 3rd, 2020 under number EK 01/2020. A copy and English translation of the original signed and dated research ethics committee approval letter has been uploaded to the journal together with the paper’s manuscript. Experiments, data collection and data storage were conducted in accordance with national and international ethical standards and, in particular, adhere to the Declaration of Helsinki and the requirements of the German Research Foundation (DFG).

### Study design

A within-subjects study design was chosen to investigate the effects of short- and long-wavelength light on the awakening response of a group of young and healthy male adults. The study was conducted under controlled experimental conditions (ambient temperature = 22.0± 1.5°C; ambient humidity = 45 ± 2%) in a sleep laboratory setting at the Pulmonary Center Darmstadt, Germany. The main experiments to illustrate the study protocol and collect the reported pilot data were carried out between May and June 2020.

One week prior to each of their individually scheduled test sessions, i.e., one for each light condition, participants were asked to keep a regular sleep-wake schedule—even on weekends—with a target sleep (wake-up) time between 11:00 pm (07:00 am) and midnight (08:00 am). Compliance was ensured by self-reported sleep diaries [56] and wearable sleep tracking using a Garmin Forerunner^®^ 945. In addition, subjects were instructed to reduce alcohol and caffeine consumption to a minimum in the week preceding the test session, while the day before they had to abstain completely.

In the evening of the day prior to their individual morning test session, the participants were required to arrive at the sleep laboratory not later than 7:00 pm. They were instructed to have dinner before at home, since no meals were provided at the test site. No specific dietary was imposed. On arrival, the participants were given the time to make themselves comfortable and prepare for the night. Starting from 8:00 pm, the light conditions in the sleep laboratory were dimmed to a minimum. This was achieved by a low-light emitting incandescent table lamp being the only source of illumination in the room. It was placed on top of a bedside table next to the subjects’ bed so that the vertical illuminance measured at the subjects’ eyes was always less than 5 lx independent of where they moved or what they did in the room. In case they wanted to go to the bathroom, which was not ensuite but on the opposite side of the hallway, subjects had to wear light-blocking glasses reducing the light of the installed luminaires to a minimum that was still acceptable for a safe orientation and movement. In particular, all blue light components were filtered out in order to prevent the risk of nocturnal melatonin suppression that might have delayed or affected sleep [57–59]. The used glasses block more than 99.9% of the light for wavelengths smaller than 500 nm and showed an average blocking coefficient of 95% over the complete visible regime, which, in our setting, gave a maximal illuminance of 10 lx at eye level. For the same reason of preventing melatonin suppression, subjects were not allowed to use any sort of self-luminous electronic devices [60, 61], such as smartphones or laptops, after 8:00 pm. However, they were permitted to read a book or listen to music and audiobooks for pastime.

Strictly controlling the light history before sleep in a way that is described here seems to be indicated not only for averting melatonin suppression and reduced sleep quality, but also with regard to the minimization of potential confounding factors that may have an effect on the next day’s morning cortisol secretion (c.f. Figueiro and Rea [28]).

**Fig 1:**
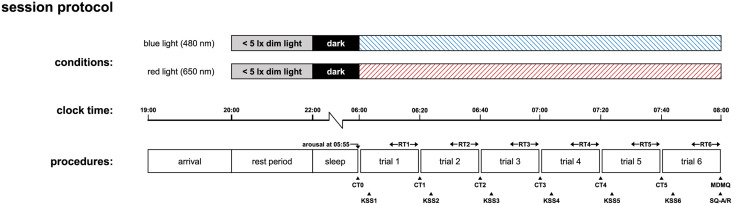
Schematic illustration of the applied within-subjects study protocol for both light conditions. Administration times for salivary cortisol sampling (CT0–CT5), auditory performance testing (RT1–RT6), and the assessment of sleepiness (KSS1–KSS6), sleep quality (SQ-A/R) and mood state (MDMQ) are explicitly indicated. Performing the experiments in an overnight sleep laboratory setting allows for strict control of the participants’ light history before sleep and ensures compliance. Participants were non-sleep-deprived as an approximate 8 h sleep opportunity was given before post-awakening light exposure.

Starting from 10:00 pm, all lights were turned off for bedtime. A 7 h and 55 min sleep opportunity was given. On the next morning, at 5:55 am, the participants were awakened by the night nurse who entered the room for preparing the collection of the first saliva sample. The sample was taken with the participants still being in bed. Afterwards, they were guided to and seated in front of an integrating sphere setup located in the same room, which was used to ensure controlled light exposure. Further details are given in the next section. Two hours of light exposure then started at 6:00 am. Saliva samples were collected every 20 min at 6:20 am, 6:40 am, 7:00 am, 7:20 am, and 7:40 am, respectively. Participants additionally rated their subjective sleepiness after each saliva sampling. Reaction times were recorded in between by applying a simple auditory vigilance task. Upon completion of the 2h test session, the participants’ current mood as well as their experienced sleep quality during the previous night were assessed using dedicated questionnaires, which are specified further down. The latter in conjunction with the data obtained from wearable sleep tracking during the overnight stay at the sleep laboratory serves to exclude the impact of poor sleep on the study results. Sleep tracking and factoranalytical analysis of subjective sleep ratings both yielded consistent results indicating that all participants experienced a regular and undisturbed pre-experimental sleep before each test session. For a better overview, the complete study protocol is summarized in Fig 1. Note that the presentation order of the two different light conditions was randomized across the subjects. The study was conducted from Monday to Friday over multiple seasons (i.e., two per subject, one for each test light condition) and was limited to one subject per day/night. For each participant, the two scheduled test sessions took place on the same day of the week while being separated by at least a period of two weeks serving as a wash-out phase.

### Experimental setup, light conditions and calibration procedure

**Fig 2:**
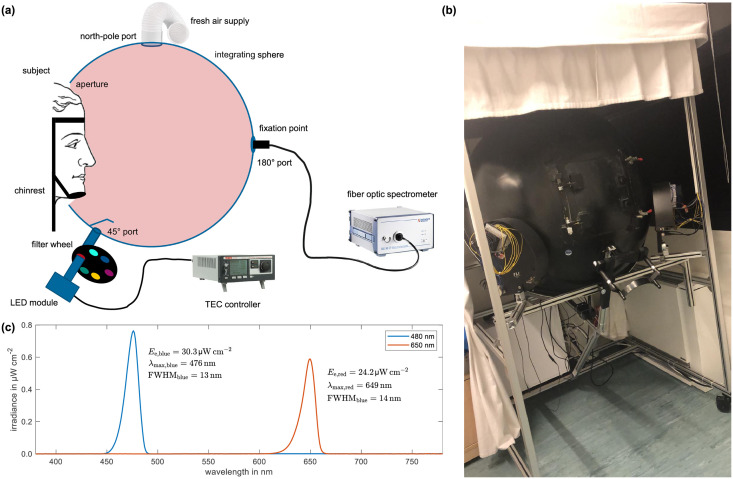
Overview of the experimental setup and test light conditions. A: Schematic illustration of the individual components of the experimental setup. Homogeneous illumination of the participants’ entire visual field is provided by using a large integrating sphere collecting the light emitted by a narrow-band LED-based temperature-stabilized light source. A fiber optic spectrometer mounted to the 180° port of the integrating sphere in combination with a calibrated photodiode placed at the participants’ right eye position is used for calibration. An integrated fresh air supply as well as the explicit control of room temperature and humidity guarantee constant ambient conditions across all test sessions. B: Image of the experimental setup as it can be found in the sleep laboratory. When not in use, the aperture door is closed with the chinrest in a stowed position to prevent dust from settling inside the sphere. C: Calibrated light spectra of both test conditions adjusted to deliver equal photon densities.

The study’s participants experienced two different experimental conditions, i.e, a 480 nm blue light (maximum wavelength λ_max_ = 476 nm, full width at half maximum FWHM = 13 nm) and a 650 nm red light (λ_max_ = 649 nm, FWHM = 14 nm) condition. Both conditions were applied using the integrating sphere setup shown in Fig 2(a) and 2(b). The integrating sphere had a diameter of 1 m and was coated with barium sulfate (ODP97, Gigahertz-Optik GmbH, Türkenfeld, Germany) on its inside, showing excellently diffuse reflection properties with a spectrally uniform reflectance of 97 ± 0.4% in the whole visible regime from 380 to 780 nm. During the 2 h light exposure of each test session, participants, while being seated in front of the integrating sphere, were instructed to look through a suitably sized aperture at the sphere’s opposite inner wall where they should focus on a small fixation point. They were asked to keep their eyes open at all times (blinking was allowed), which was monitored by the experimenters. For convenience and in order to ensure proper light exposure and alignment, participants were further instructed to put their head on a dedicated chinrest that was attached to the metal frame holding the integrating sphere. In addition, fresh air supply was provided by a fan installation with integrated air-filtering, which was mounted to the sphere’s north-pole port.

The light sources delivering both experimental conditions were realized by combining a Peltier-cooled LED module comprising an array of either blue or red LEDs with a filter wheel attached to it. Both light sources were then mounted to the sphere’s ± 45° ports on the left and right hand side of the aperture (c.f., Fig 2(b)). The LEDs were driven in constant current mode to avoid flicker and cooled to about 35°C for achieving high efficacy. The used narrow-band interference filters were chosen such that their transmittance profiles matched with the LEDs’ peak wavelengths. Subsequent calibration was performed in order to assure equal photon densities for both light conditions.

For this purpose, a calibrated silicon photodiode (SM05PD1A, Thorlabs, Newton, NJ, USA) connected to a power meter (PM100USB, Thorlabs, Newton, NJ, USA) for readout was placed in the vertical plane at the participants’ approximate right-eye position when using the chinrest. Knowing the size of the active sensor area thus allowed for measuring the resulting irradiance as a function of LED current. In addition, a wavelength-calibrated fiber optic spectrometer (CAS 140 B, Instrument Systems Optische Messtechnik GmbH, Munich, Germany) attached to the 180° port of the integrating sphere was used to monitor the spectral behavior. Hence, by knowing both the absolute irradiance *E*_e_ and the relative spectral composition of the incident light *E*_rel,λ_ at the participants’ eye level, the corresponding photon density *ϱ*_ph_ could be calculated using

$$\begin{array}{c} \varrho_{\text{ph}}=\int_{380\;\text{nm}}^{780\;\text{nm}}\;\frac{E_{\text{e},\lambda }}{E_{\text{ph}}}\;\text{d}\lambda =\int_{380\;\text{nm}}^{780\;\text{nm}}\;\frac{a\cdot E_{\text{rel},\lambda }}{E_{\text{ph}}}\;\text{d}\lambda , \end{array}$$ (1)

where *E*_e,λ_ = d*E*_e_/dλ is the spectral irradiance and *E*_ph_ = *hc*/λ is the photon energy at wavelength λ with *h* being the Planck constant and *c* the speed of light in vacuum. The relation between absolute and relative measures is further given by the calibration factor *a* that is obtained from

$$\begin{array}{c} a=\frac{E_{\text{e}}}{\int_{\;\;\;\;380\;\text{nm}}^{\;780\;\text{nm}}\;E_{\text{rel},\lambda }\;\text{d}\lambda }. \end{array}$$ (2)

Thus, by varying the current used for driving the blue and the red light LEDs accordingly, both light conditions, shown in Fig 2(c), were adjusted to deliver 7.58 ± 0.44 × 10^13^ photons cm^−2^ s^−1^. This corresponds to an approximate vertical illuminance at the participants’ corneas of 24 ± 3 lx for the blue and of 22 ± 2 lx for the red light condition, which was measured using an illuminance meter (HCT-99D, Gigahertz-Optik GmbH, Türkenfeld, Germany). Note that these intensity values serve only as an example in a range that can be expected to be effective according to the findings of e.g. Brainard *et al*. [42] and Thapan *et al*. [43] causing mid- to up-range saturation for melatonin suppression. However, for the proper determination of a dose-response relationship, considerably larger (and also smaller) intensities have to be probed to capture the complete picture.

In order to obtain estimates for the retinal responses to be expected for the current settings, corresponding *α*-opic irradiances were calculated. This was done by weighting each spectrum with the spectral sensitivity of each of the five different photoreceptors (L-, M-, and S-cones, rods, and melanopsin-encoded ipRGCs) and integrating the weighted spectrum over all bands of wavelength between 380 nm and 780 nm [62, 63]. The spectral sensitivities used for performing these calculations were extracted from the recently published CIE standard CIE S 026/E:2018 [64] and the results are summarized in Table 1.

**Table 1:** Comparison of the two test light conditions in terms of irradiance, photopic illuminance, and *α*-opic irradiances of the individual retinal photoreceptors, i.e., S-, M-, L-cones, rods, and ipRGCs.

measure	unit	480 nm blue light	650 nm red light
S-cone-opic irradiance	μW cm^−2^	15.31	0.03
M-cone-opic irradiance	μW cm^−2^	9.12	0.55
L-cone-opic irradiance	μW cm^−2^	5.31	4.65
rhodopic irradiance	μW cm^−2^	21.52	0.06
melanopic irradiance	μW cm^−2^	26.69	0.03
irradiance	μW cm^−2^	30.29	24.24
illuminance	lx	23.31	22.91

### Saliva collection and cortisol analysis

Saliva samples for the determination of post-awakening cortisol levels were collected according to the session protocol shown in Fig 1 using the Sarstedt Salivette^®^ Cortisol system (SARSTEDT AG & Co. KG, Nümbrecht, Germany). During each test session, six saliva samples (one reference right after awaking and five follow-ups every 20 min) were obtained per subject. After collection, the samples were immediately stored in the fridge at 4°C. At the end of each test session, the samples were prepared for shipping using insulated boxes and thermal packs, as the cortisol assays were outsourced to a specialized analysis laboratory (daacro GmbH & Co. KG, Trier, Germany).

Double-determinations of the cortisol concentration in the saliva samples were performed by applying competitive enzyme-linked immunosorbent assay (ELISA) analysis (Salimetrics, State College, PA, USA), which offers a sensitivity for cortisol detection of 0.19 nmol L^−1^. The mean values of these double-determinations were subsequently used for the pilot analysis presented in this work. Corresponding intra- and inter-assay coefficients of variation (CVs) were 2.72% and 2.38%, respectively.

### Assessment of subjective sleepiness, mood, and previous night’s sleep quality

Data of subjective sleepiness, mood, and sleep quality were obtained from the participants through a combination of interview-based and self-administered questionnaires following the session protocol of Fig 1. Subjective sleepiness was assessed by using the Karolinska Sleepiness Scale (KSS) developed by Åkerstedt and Gillberg [65]. This verbally anchored nine-point rating scale measures the participants’ feeling of current sleepiness ranging from 1 = “extremely alert” to 9 = “extremely sleepy, fighting sleep”. KSS ratings were collected five minutes after each saliva sampling in an interview-based manner conducted by the experimenters, so that six ratings were obtained per experimental session. In order to compare the overall trend in the ratings of subjective sleepiness between the different test conditions, mean values were calculated from the six individual ratings of each experimental session.

At the end of each experimental session, participants were further asked to rate their current mood state as well as their experienced sleep quality during the previous night using self-administered questionnaires. Several dimensions of mood were assessed at the same time by adopting the multi-dimensional mood questionnaire (MDMQ) developed by Steyer *et al*. [66]. It consists of 24 mood-defining adjectives (items) that correspond to one of following three dimensions: i) Good-vs-bad mood, ii) awake-vs-tired, and iii) calm-vs-nervous, where each pole is encoded by four different items. For each of these items, participants should express their agreement or disagreement on a bipolarly conceptualized five-step rating scale, where 1 = “not at all” and 5 = “very”. Each mood dimension is then analyzed separately. For this purpose, the ratings for the items representing the negative mood poles must be inverted so that they can be summed up together with the ratings for the items representing the positive mood poles. This resulted in a single metric for each mood dimension ranging from 8 (minimum) to 40 (maximum), where a high value indicates a positive mood state with regard to the respective subscale.

Sleep quality during the previous night was measured by applying the revised (fourth) edition of the sleep questionnaire (SF-A/R) developed by Görtelmeyer [67, 68]. It comprises 25 questions addressing both quantitative (bedtime, sleep onset, wake-up time, frequency and duration of sleep interruptions) and qualitative (sleeping behavior, previous day events, somatic symptoms during sleep, general sleep rating, mood state before and after sleep) aspects of sleep. The participants ratings, time designations, and frequency indications are then used to derive representative values on five factoranalytical subscales representing the factors i) general sleep quality (SQ), ii) feeling of recovery after sleep (RAS), iii) mental balance before sleep (MBBS), iv) psychic fatigue before sleep (PFBS), and v) psychosomatic symptoms during sleep (PSS). Corresponding values always range from 1 (minimum) to 5 (maximum) and their interpretation must be performed according to the polarity of the respective subscale. That is, a value close to the upper limit on the SQ, RAS, and MBBS scales indicate that the subject experienced good-quality sleep, showed a positive feeling of recovery, and was in a good mood state before going to bed. Regarding on the other hand the PFBS and PSS factors, a large score suggests that the subject suffered from an enhanced psychic fatigue before sleep and an increased burden of psychosomatic symptoms during sleep.

### Auditory vigilance task

A simple auditory vigilance task (AVT) [69, 70] was applied to measure reaction times. The corresponding soft- and hardware implementation was developed using the PsychoPy package [71] (version 3.0.7) in conjunction with a USB-tethered Microsoft Xbox One controller enabling excellent precision in stimulus presentation and response timing with very low latencies [72]. All components were installed on a computer running Windows 10 as the operating system. Following the session protocol shown in Fig 1, six consecutive AVTs were administered to the participants over the course of the second half of each session block (trial). Each of these tests lasted 10 min, in which participants had to respond to a sequence of auditory stimuli by pushing the right shoulder button (bumper button RB) of the Xbox controller as soon as they hear a sound signal that was provided over the computer’s on-board audio interface using wired noise-canceling headphones (Bose QuietComfort 35 II, Bose Corporation, Framingham, MA, USA). The sound signals are generated based on the low-latency Psychophysics Toolbox [73] (PTB) audio library called directly from PsychoPy. These signals showed a frequency of 1 kHz and were presented to the participants for exactly 100 ms. The inter-stimulus interval was randomly generated to take a value between 4 s and 10 s and balanced in such a way that each trial sequence consisted of 93 stimuli. The volume was adjusted prior to the study for the sound signals to be easily audible.

Participants’ AVT responses were considered as valid in case that the corresponding reaction times (RTs) were larger than the commonly acknowledged threshold for stimulus perception and human motor response of 150 ms [74]. In addition, lapses were defined and excluded from the analysis for RTs lying more than 1.5 interquartile ranges above the upper quartile of each trial sequence. RT recording started instantaneously with the stimulus presentation.

## Pilot data

This section is intended to report on some pilot data that were collected following the proposed study protocol. The test panel of subjects, as described in the “Materials and methods” section, comprised a total of four healthy men meeting the study criteria. Corresponding mean values of the various outcome measures for both lighting scenarios are thus summarized in Table 2, allowing for a trend analysis.

**Table 2:** Mean±SEM results of the various physiological and subjective outcome measures.

measure	unit	480 nm blue light	650 nm red light
mean cortisol levels	nmol L^−1^	12.04 ± 1.54	9.68 ± 1.21
mean reaction times	ms	393.57 ± 57.76	402.05 ± 60.05
subjective sleepiness	-	4.54 ± 0.39	5.46 ± 0.29
good-vs-bad mood	-	34.25 ± 2.02	34.25 ± 1.12
awake-vs-tired	-	25.75 ± 2.95	26.50 ± 2.75
calm-vs-nervous	-	30.75 ± 2.29	31.25 ± 1.97

As can be seen, the overall mean of the measured salivary cortisol concentrations is about 24% larger for the blue light than for the red light condition. Being in accordance with existing literature, this finding indicates that exposure to short-wavelength light in the morning hours may provoke an enhanced post-awakening cortisol secretion, whereas no or at least a less pronounced enhancement is observed for its long-wavelength counterpart. Similarly, mean ratings of subjective sleepiness are reduced by 1 KSS score point for the blue light compared to the red light condition, which suggests that, within the 2-hours test period, subjects overall felt less sleepy during the short-wavelength intervention. No differences on the other hand could be observed for the mean reaction times and mood ratings.

In order to exclude the impact of poor sleep on the study results, data of wearable sleep tracking as well as subjective ratings of the SQ-A/R questionnaire were collected from each participant. In all cases, the subjects’ total sleep duration was 7 h or more. In addition, sleep data did not show any evidence for longer episodes of nocturnal arousals, other sleep disturbances, or an awakening before the scheduled wake-up time. On average, subjects felt asleep within the first 30 min after turning off the lights for bedtime. Furthermore, separate visual inspection of the different subscales of the SQ-A/R questionnaire revealed that, across all test sessions, participants were always in a good mood and mental state before going to bed, experienced a regular, undisturbed, good-quality sleep and showed a positive feeling of recovery. No obvious differences were found between the test conditions, neither on any of the SQ-A/R subscales nor for the collated wearable sleep tracking data. Thus, sleep tracking and the analysis of subjective sleep ratings both yielded consistent results that allow for excluding potential confounding factors related to poor sleep.

## Discussion and future directions

The present work proposes a methodological framework in form of a study protocol to explore the spectral dependencies of the post-awakening effects of 2-hours of monochromatic light exposure on the CAR and potentially related, secondary outcome measures. A protocol summary (S1 File) is available for download and can be accessed from the Supporting information section of this paper. As a proof of concept, the discussed study protocol was applied to collect pilot data of cortisol levels, reaction times as well as subjective ratings of mood state and sleepiness for a group of four healthy, non-sleep-restricted, male adults. As part of these experiments, which were conducted under controlled laboratory conditions, each participant was exposed to two different wavelength settings of short-wavelength blue light at λ_max_ = 476 nm and long-wavelength red light at λ_max_ = 649 nm, respectively.

The reported pilot data, even though strongly limited due to small sample sizes, are in line with previous findings and confirm the stimulatory effect of post-awakening light exposure on the CAR. Comparing both tested light conditions, blue-light exposure was associated with higher post-awakening cortisol levels than red-light exposure. This basically is in accordance with previous data from Petrowski *et al*. [38], who also reported an increased positive effect towards higher cortisol levels for blue (and green) light, while observing a significantly weaker response for red light. Thus, it seems to be likely that the magnitude of post-awakening light-induced effects on cortisol secretion is most sensitive for short-wavelengths. A potential relation to the melatonin pathway, which is mediated by retinal projections from a subset of melanopsin-expressing retinal ganglion cells (mRGCs) to the SCN, has been discussed in the literature [28, 38]. Despite some first clues pointing towards this direction (further encouraged by the results of the current work), additional research is still required for drawing an ultimate conclusion.

Regarding the additional outcome measures considered in this work, it was found that blue-light compared to red-light exposure tended to improve the subjective perception of sleepiness and mood ratings within the 2-hours test period, although the absolute difference of mean ratings was quite small between both light conditions. Furthermore, no considerable differences were observed with regard to reaction times and mood state. Thus, these results seem to be in contrast to the initial assumption that the observed blue-light-induced enhancement in the CAR (i.e., the “boosting”) may also translate to acute measures of cognitive performance and mood. With evidence being provided that long-wavelength red light evokes acute alerting effects and impacts mood state via non-endocrine pathways mediated by L-cones [75–81], the present results in fact indicate that neuroendocrine and neurobehavioral post-awakening responses to light should be considered separately. While the endocrine pathways involved in the regulation of morning cortisol secretion seem to be most sensitive for short wavelengths, the spectral sensitivities of non-endocrine pathways to morning light exposure are likely to show rather different characteristics. Supported by the pilot data of the present study, an equal efficacy must be assumed for both short- and long-wavelength light exposure with regard to mediating acute post-awakening effects on cognitive and emotional functioning. Thus, no evidence is given for a correlation between CAR enhancement and the measures of mood state and performance considered during the two hours post awakening. Nevertheless, it is still possible that the potential boosting effect of an enhanced CAR might be carried over to a later point in time after the actual light exposure. In any case, further empirical evidence is needed including the derivation of spectral sensitivity functions for alertness and other non-endocrine daytime responses. Functional magnetic resonance imaging (fMRI) and electroencephalographic (EEG) measurements may help to identify corresponding neural pathways.

Limitations of the reported results based on the collected pilot data—even though in accordance with the literature—are mainly related to the rather low number of participants included so far. Due to budgetary constraints it was not yet possible to extend the proposed methodology to a larger sample size, which would obviously yield a strongly reduced power when attempting to perform any statistical analysis on that data. Nevertheless, as the main purpose of this lab protocol is to summarize the proposed methodology for a consistent exploration of the spectral dependencies of CAR and other potentially related outcomes, the present work clearly paves the way for future exploratory research in that field.

Apart from the rather low number of participants, the interpretation of the results of the present study is further limited by the fact that, also due to budgetary constraints, no dim light control was included so that no inferences in relation to a reference condition can be drawn. Future research on the spectral dependencies of CAR and potentially related phenomena must include such a reference to fully elucidate the effects of post-awakening light exposure. In addition, generalizability is deflated as only male subjects of normal chronotype belonging to a specific age group were considered for participating in the experiments in order to minimize potential confounding factors. However, literature suggests that chronotype, gender, and age all essentially influence CAR [21, 26, 27, 82–85] and, as such, should explicitly be included in future studies with considerably larger sample sizes to investigate how these factors may differentially interact with the light-induced effects on morning cortisol secretion. Moreover, it should be noted that the targeted bed and wake-up times for collecting the pilot data reported in this work are rather early for non-early chronotypes so that an individual adaptation of the experimental schedule might be necessary to better account for variations in the circadian rhythms between different subjects. For collecting the pilot data, we were strictly limited by the regular operating hours (starting from 9:00 am) of the Pulmonary Center Darmstadt, where the sleep laboratory experiments took place, so that all procedures had to be completed not later than 8:30 am.

Further methodological improvements in the study design can be achieved by extending its general protocol by an acclimatization period for the participants to get used to the sleep laboratory environment. Even though the use of a randomized design in combination with sleep quality monitoring reduces the probability for systematic errors influencing the study results, the inclusion of one or more acclimatization nights might be favorable for consolidating the participants sleep-wake pattern to be in accordance with the study protocol and additionally ensures the complete wash-out of potential confounding effects of previous light history. Finally, with the current experimental setting, effects of orthostatic changes when subjects had to move from lying in bed to sitting in front of the experimental device for light exposure cannot be precluded. Thus, the adoption of more flexible approaches for light application, such as light goggles or miniaturized ganzfeld spheres applied to each of the subject’s eye, that can effectively be used to deliver the desired lighting conditions while the subjects can remain in their initial posture, may help to exclude such potentially confounding factors.

## Conclusion

Success in determining the spectral dependencies of morning cortisol secretion and potentially related outcome measures in response to light stimuli of different spectral composition and intensity—as it was performed for the melatonin pathway expressed in terms of nocturnal melatonin suppression [86]—serves as an appropriate starting point for the development of a daytime-based model of how different light affects non-visual responses in the human organism during the waking hours. Understanding the underlying dose-response relationships further elucidates the involved processes of human circadian phototransduction. From a practical point of view, the development of such a model may eventually allow for the derivation of an advanced planning and decision metric in the context of integrative (human-centric) lighting [87–91]. In this context, the current lab protocol provides an overview of the proposed methodology to explore the corresponding spectral dependencies and to discuss measures of emotional state and cognitive functioning potentially related to the CAR in a consistent and expedient manner under controlled laboratory conditions. This document has thus been prepared to help other researchers in the field to develop and improve their own approaches for investigating these interactions. We therefore do not intend to implement the protocol any further.

## Supporting information

S1 File Study protocol summary. An additional lab protocol summarizing the methodology proposed in this work is accessible from https://dx.doi.org/10.17504/protocols.io.btxanpie.
(PDF)
