# Supplementary material for: Study protocol for measuring the impact of (quasi-)monochromatic light on post-awakening cortisol secretion under controlled laboratory conditions
Source: PLoS One. 2022 May 18;17(5):e0267659. doi: 10.1371/journal.pone.0267659 (PMC9116651; doi:10.1371/journal.pone.0267659)
Supplement: S1 File — An additional lab protocol summarizing the methodology proposed in this work is accessible from https://dx.doi.org/10.17504/protocols.io.btxanpie. (PDF) [file pone.0267659.s001.pdf]

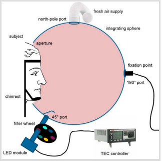

Lab protocol for assessing the spectral dependencies of the cortisol awakening response (CAR) and potentially related outcome measures for morning light exposure

Sebastian Babilon[1,2], Paul Myland[1], Julian Klabes[1], Joel Simon[1], Tran Quoc Khanh[1]

[1]Laboratory of Lighting Technology, Technical University of Darmstadt, Hochschulstr. 4a, 64289 Darmstadt, Germany;
[2]Light and Health Research Center, Department of Population Health Science and Policy, Icahn School of Medicine at Mount Sinai, One Gustave L. Levy Place, New York, NY 10029, USA
Sebastian Babilon: babilon@lichttechnik.tu-darmstadt.de;

dx.doi.org/10.17504/protocols.io.btxanpie

Sebastian Babilon

ABSTRACT

Cortisol secretion has a fundamental role in human circadian regulation. The cortisol awakening response (CAR) can be observed as a daily recurring sharp increase in cortisol concentration within the first hour after awakening and is influenced by environmental light conditions. However, so far, the spectral dependencies of this process are still largely unknown. This research protocol therefore aims at establishing a fundamental methodology that allows for the exploration of the spectral dependencies of the CAR in a consistent and expedient manner. A concomitantly published laboratory report paper presents preliminary results applying this methodology and creates the base for future research on the spectral dependencies of CAR and potentially related outcome measures of emotional state and cognitive functioning.

THIS PROTOCOL ACCOMPANIES THE FOLLOWING PUBLICATION

S. Babilon et al. Short-wavelength light compared to long-wavelength light enhances post-awakening cortisol secretion, submitted for publication

DOI

dx.doi.org/10.17504/protocols.io.btxanpie

PROTOCOL CITATION

Sebastian Babilon, Paul Myland, Julian Klabes, Joel Simon, Tran Quoc Khanh . Lab protocol for assessing the spectral dependencies of the cortisol awakening response (CAR) and potentially related outcome measures for morning light exposure. **protocols.io** https://dx.doi.org/10.17504/protocols.io.btxanpie

MANUSCRIPT CITATION please remember to cite the following publication along with this protocol

S. Babilon et al. Short-wavelength light compared to long-wavelength light enhances post-awakening cortisol secretion, submitted for publication

KEYWORDS

cortisol awakening response, morning light exposure, spectral dependencies, cognitive functioning, mood state, sleepiness

LICENSE

This is an open access protocol distributed under the terms of the Creative Commons Attribution License, which permits unrestricted use, distribution, and reproduction in any medium, provided the original author and source are credited

CREATED

Apr 03, 2021

Citation: Sebastian Babilon, Paul Myland, Julian Klabes, Joel Simon, Tran Quoc Khanh ( ). Lab protocol for assessing the spectral dependencies of the cortisol awakening responseÃÂ (CAR) and potentially related outcome measures for morning light exposure. https://dx.doi.org/10.17504/protocols.io.btxanpie

LAST MODIFIED

Apr 10, 2021

PROTOCOL INTEGER ID

48834

MATERIALS TEXT

Experimental Setup and Calibration

1. Integrating sphere, barium sulfate coated, diameter of 1 m, suitable aperture to homogeneously illuminate the entire visual field of the subjects, dedicated ports for light source and fiber optic spectrometer, fresh air supply
2. Peltier-cooled temperature-stabilized LED light source with monochromatic LED modules
3. Filter wheel with interference filters
4. Calibrated silicon photodiode (e.g., SM05PD1A, Thorlabs, Newton, NJ, USA) incl. power meter for read-out (e.g., PM100USB, Thorlabs, Newton, NJ, USA)
5. Wavelength-calibrated fiber optic spectrometer (e.g., CAS 140 B, Instrument Systems Optische Messtechnik GmbH, Munich, Germany)
6. CIE standard CIE S 026/E:2018 for α -opic calculations

Participant selection, data acquisition, and compliance (before and during study sessions)

1. PSQI
2. MCTQ
3. SF-36
4. PSS-10
5. Ishihara's Tests for Colour Deficiency: 38 Plates Edition,
6. Standard Pseudoisochromatic Plates Part II for Acquired Color Vision Defects
7. Farnsworth-Munsell D-15 Color Vision Test
8. Self-reported sleep diary
9. Actigraphy / wearable sleep tracking (e.g., Garmin Forerunner® 945)
10. SF-A/R
11. KSS
12. MDMQ
13. Auditory reaction time task
14. Sarstedt Salivette® Cortisol collection system or comparable
15. ELISA kits for determination of cortisol concentration
16. Fridge for saliva storage
17. Light blocking glasses

Experimental setup, light conditions, and calibration procedure

1 Proper application of light stimulus is crucial. Circadian phototransduction suggests that all retinal receptors are involved for non-image forming processes. As a consequence, illumination of the subjects' complete visual field of view is required by using an integrating sphere approach (see Sec. 1.1). Fig. 1 summarizes the suggested experimental setup and illustrates the measurement equipment for calibration purposes.

Citation: Sebastian Babilon, Paul Myland, Julian Klabes, Joel Simon, Tran Quoc Khanh ( ). Lab protocol for assessing the spectral dependencies of the cortisol awakening responseÃÂ (CAR) and potentially related outcome measures for morning light exposure. https://dx.doi.org/10.17504/protocols.io.btxanpie

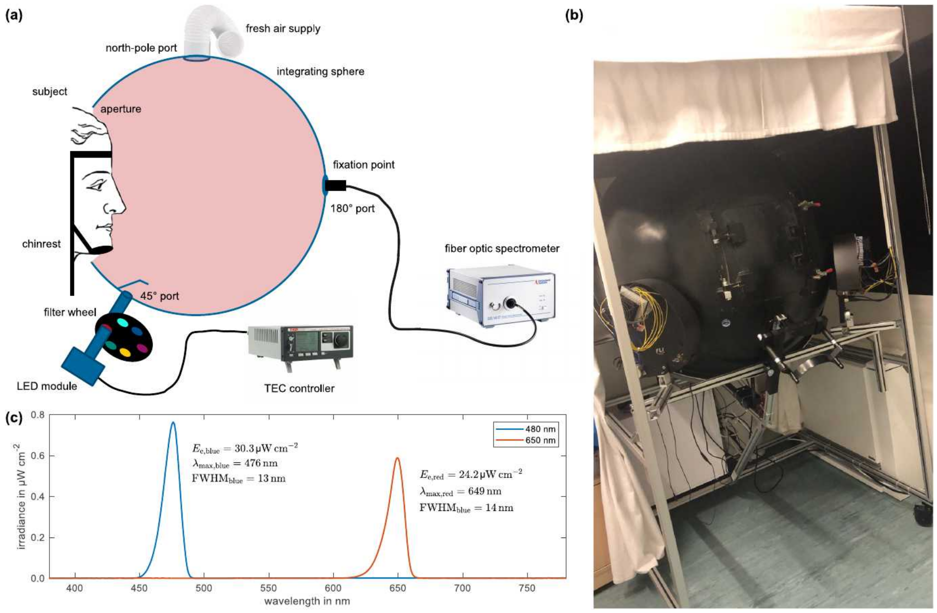

Fig. 1: Illustration of the proposed experimental setup. A: Schematic overview. Homogeneous illumination of the participants' entire visual field is crucial and is provided by using a large integrating sphere with a diameter of 1 m, collecting the light emitted by a narrow-band LED-based temperature-stabilized light source. A fiber optic spectrometer mounted to the 180° port of the integrating sphere in combination with a calibrated photodiode placed at the participants' right eye position is used for calibration. An integrated fresh air supply as well as the explicit control of room temperature and humidity guarantee constant ambient conditions across all test sessions. The subjects' head is held in position by a dedicated chin rest. **B:** Image of the experimental setup as it can be found in our laboratory. **C:** Examples of calibrated light spectra used to run the proof-of-concept study, whose results and data will be published alongside this lab protocol.

Thorough calibration of light stimuli at the subjects' eye level is required (see Sec. 1.3). Light stimuli shall be provided by a narrow-band temperature-stabilized LED light source and monitored by a fiber optic spectrometer both attached to dedicated ports of the integrating sphere (see Sec. 1.2).

1.1 **Integrating sphere:** Integrating sphere should be large enough, ~1 m of diameter recommended, for the subjects to feel comfortable and non-constricted. Fresh air supply must be ensured to prevent increasing fatigue as a confounding factor because of reduced oxygen. The integrating sphere shall be coated with barium sulfate on its inside to offer excellently diffuse reflection properties of spectral uniformity in the visible range. For convenience and in order to ensure proper light exposure and subject alignment, the subjects' head should be fixated by a comfortable chin rest.

1.2 **Light source:** The narrow-band light source is proposed to consist of Peltier-cooled monochromatic LED modules (one module of sufficient radiation output for each wavelength condition to be tested) in combination with narrow-band interference filters, whose spectral transmittance profiles are matched to the LED modules' peak wavelengths. To avoid flicker, LEDs should be driven in constant current mode. In addition, they should be cooled down and stabilized to 35°C for high radiation efficacy.

1.3 ⚠

Calibration of light stimuli: To evaluate the effect of wavelength on CAR and potentially related outcome measures, light stimuli must be adjusted to equal photon densities. A calibrated photodiode should be placed in the vertical plane at the participants' approximate right-eye position when using the chin rest. In addition, a wavelength-calibrated fiber optic spectrometer attached to a dedicated port of the integrating sphere is needed to monitor the spectral behavior. The following calibration steps are required:

1. Use photodiode to measure absolute irradiance E_e at the subjects' right eye position for a given LED current

Citation: Sebastian Babilon, Paul Myland, Julian Klabes, Joel Simon, Tran Quoc Khanh ( ). Lab protocol for assessing the spectral dependencies of the cortisol awakening responseÃÂ (CAR) and potentially related outcome measures for morning light exposure. https://dx.doi.org/10.17504/protocols.io.btxanpie

2. At the same time, measure the relative spectral composition $E_{\mathrm{rel},\lambda}$ of the light stimulus provided by the integrating sphere using the fiber optic spectrometer.
3. Calculate the corresponding photon density ϱ_{ph} using $\varrho_{\mathrm{ph}} = \int_{380\ \mathrm{nm}}^{780\ \mathrm{nm}} \frac{E_{\mathrm{e},\lambda}}{E_{\mathrm{ph}}}\,\mathrm{d}\lambda = \int_{380\ \mathrm{nm}}^{780\ \mathrm{nm}} \frac{a \cdot E_{\mathrm{rel},\lambda}}{E_{\mathrm{ph}}}\,\mathrm{d}\lambda$, where $E_{\mathrm{e},\lambda} = \mathrm{d}E_{\mathrm{e}}/\mathrm{d}\lambda$ is the spectral irradiance and $E_{\mathrm{ph}} = hc/\lambda$ is the photon energy at wavelength λ with h being the Planck constant and c the speed of light in vacuum.

   The relation between absolute and relative measures is further given by the calibration factor a that is obtained from $a = E_{\mathrm{e}} / \int_{380\ \mathrm{nm}}^{780\ \mathrm{nm}} E_{\mathrm{rel},\lambda}\,\mathrm{d}\lambda$.
4. Vary LED current and re-perform calibration / measurement procedure until the desired photon density is reached
5. Repeat for all wavelengths / LED modules to be tested and choose individual driving currents such that equal photon densities are ensured between conditions
6. Report on expected retinal responses by calculating α -opic irradiances (L-, M-, S-cone, rods, and melanopsin-encoded ipRGC exitance) according to CIE standard CIE S 026/E:2018.

Participants

2 Subject eligibility

The following questionnaires / tests are recommended to be used for proper subject selection:

1. Pittsburgh Sleep Quality Index, PSQI
2. Munich Chronotype Questionnaire, MCTQ
3. 36-item Short-Form Health Survey, SF-36
4. 10-item Perceived Stress Scale, PSS-10
5. Screening for color deficiency using Ishihara's Tests for Colour Deficiency: 38 Plates Edition, the Standard Pseudoisochromatic Plates Part II for Acquired Color Vision Defects, and the Farnsworth-Munsell D-15 Color Vision Test

Candidates shall be excluded if they showed a PSQI score of five or greater, were identified as extreme morning or evening types from the MCTQ, had scores on any of the SF-36 sub-scales that were at least one standard deviation smaller than the respective age- and gender-dependent mean values of the normative sample obtained from the German Federal Health Survey 1998, experienced recent episodes of intense stress indicated by a PSS-10 score of more than one standard deviation larger than the age- and gender-dependent mean score of a representative sample of the German population, or showed indication for any sort of color vision impairments

In addition, experimenters should check for (personal interview) and exclude

1. Smoking
2. Medication or drug consumption
3. excessive alcohol use or caffeine intake
4. history of chronic health problems
5. psychiatric, mental, or sleep disorders
6. (late / night) shift work or transmeridian flights within the last two months prior to the screening day and / or test session

Candidates wearing glasses or contact lenses for visual acuity correction do not need to be excluded from the study as long as the limits of ± 6 dpt or ± 4 dpt in case of astigmatism were not exceeded. However, they must be asked to remove their optical aids before beginning with the light exposure session.

Citation: Sebastian Babilon, Paul Myland, Julian Klabes, Joel Simon, Tran Quoc Khanh ( ). Lab protocol for assessing the spectral dependencies of the cortisol awakening responseÃÂ (CAR) and potentially related outcome measures for morning light exposure. https://dx.doi.org/10.17504/protocols.io.btxanpie

Potential confounding factors such as gender or age must either be controlled, e.g., by selecting only (fe-)males of a certain age to take part in the experiments, or explicitly included in the analysis. Note that for the latter, sufficient statistical power must be ensured. Power analysis should be performed to estimate appropriate sample size.

Subject compliance for minimizing confounding factors

In order to minimize potentially confounding factors, all subjects must adhere to a regular sleep-wake schedule the week before each of their individual test sessions and reduce their alcohol and caffeine consumption to a minimum. Recommendations are:

1. target sleep time at 11:00 pm ± 1 h
2. target wake-up time 07:00 am ± 1h
3. less than 60 g of pure alcohol per week (~ 1.5 l of beer with an ABV of 5%)
4. max. 200 mg caffeine per day (~ 300 ml of black coffee)

The day before each of their test sessions, subjects should abstain completely. Compliance to regular sleep-wake pattern should be controlled based on self-reported sleep diaries and wearable sleep tracking / actigraphy.

Study design and data collection

3 22.0 °C 45 % recommended environmental conditions

Fig. 2 shows the proposed study design and data collection procedure to evaluate the effect of wavelength on CAR and related outcome measures.

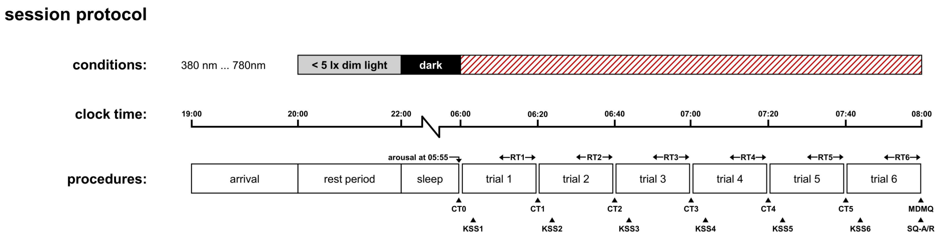

Fig. 2: Proposed session protocol for data acquisition. Recommended administration times for salivary cortisol sampling (CT0-CT5) are explicitly indicated. Additionally shown are sampling times for auditory reaction time testing (RT1-RT6), assessment of sleepiness (KSS1-KSS6), and acquisition of mood state (MDMQ) representing potentially related outcome measures considered as examples in the concomitantly published laboratory report paper. Assessment of sleep quality during the previous night (SF-A/R) serves compliance purposes.

Data collection should be performed under controlled experimental conditions in a sleep laboratory setting. The evening prior to each of their individual morning test sessions (one for each wavelength to be tested for within-subjects study design), the participants are required to arrive at the sleep laboratory not later than 7:00 pm to get prepared for the night.

Starting from 8:00 pm, the light conditions in the sleep laboratory must be dimmed to a minimum (< 5 lx at vertical eye level) to prevent the risk of nocturnal melatonin suppression that may delay or negatively affect sleep. Light blocking glasses should be worn in case dimming of room illumination to low values is not possible. No electronic self-luminous devices, such as smartphones or laptops, are permitted after 8:00 pm.

Sleep opportunity starting at 10:00 pm with all lights being turned off. Wake-up of participants is initiated the next morning at 5:55 am with the first saliva sample being collected while they are still in bed, which gives the reference / baseline level of cortisol concentration at the beginning of each test condition. Two hours of light exposure at given wavelength test condition then start right after at 6:00 am. Saliva samples should be collected at least every 20 min. If budget allows more frequent sampling is favorable (cf. Stalder et al.). Ratings / Assessments of other outcome measures acquired in parallel should be collected at equidistant sampling times between two subsequent saliva samples. In the concomitantly published laboratory report paper, intended to present preliminary study results applying the methodology summarized in this protocol, these are the assessment of simple auditory reaction times, subjective sleepiness, KSS, and current mood state, MDMQ.

Citation: Sebastian Babilon, Paul Myland, Julian Klabes, Joel Simon, Tran Quoc Khanh ( ). Lab protocol for assessing the spectral dependencies of the cortisol awakening responseÃÂ (CAR) and potentially related outcome measures for morning light exposure. https://dx.doi.org/10.17504/protocols.io.btxanpie

Note: Actigraphy / sleep tracking during overnight stay in combination with the SF-A/R sleep quality questionnaire ensures compliance (e.g., exclusion of the impact of poor sleep or a too early wake-up before the scheduled time) and allows for including sleep quality and duration as potentially confounding factors in the statistical analysis of the data and the model building. Order of presentation of the different wavelength test conditions should be randomized across subjects.

Saliva samples should be collected using the Sarstedt Salivette® Cortisol system or comparable for the ease of use. After collection, the samples should immediately stored in a fridge at about **4 °C** and, despite a certain robustness of saliva cortisol, analyzed as fast as possible. Double-determination of cortisol concentration is recommended using competitive enzyme-linked immunosorbent assay (ELISA) analysis.

Citation: Sebastian Babilon, Paul Myland, Julian Klabes, Joel Simon, Tran Quoc Khanh ( ). Lab protocol for assessing the spectral dependencies of the cortisol awakening responseÃÂ (CAR) and potentially related outcome measures for morning light exposure. https://dx.doi.org/10.17504/protocols.io.btxanpie
